# Structural features of chloroplast trigger factor determined at 2.6 Å resolution

**DOI:** 10.1107/S2059798322009068

**Published:** 2022-09-27

**Authors:** Yvonne Carius, Fabian Ries, Karin Gries, Oliver Trentmann, C. Roy D. Lancaster, Felix Willmund

**Affiliations:** a Department of Structural Biology, Saarland University, Center of Human and Molecular Biology (ZHMB), Faculty of Medicine, Building 60, 66421 Homburg, Germany; b Molecular Genetics of Eukaryotes, University of Kaiserslautern, Erwin-Schrödinger-Strasse 70, 67663 Kaiserslautern, Germany; c Molecular Botany, University of Kaiserslautern, Erwin-Schrödinger-Strasse 70, 67663 Kaiserslautern, Germany; University of Vienna, Austria

**Keywords:** molecular chaperones, chaperone trigger factor, chloroplasts, *Chlamydomonas reinhardtii*, co-translational folding, PPIases

## Abstract

The folding of newly synthesized polypeptides requires the coordinated action of molecular chaperones. Prokaryotic cells and the chloroplasts of plant cells possess the ribosome-associated chaperone trigger factor, which binds nascent polypeptides at their exit stage from the ribosomal tunnel. The structure of bacterial trigger factor has been well characterized and it has a dragon-shaped conformation, with flexible domains responsible for ribosome binding, peptidyl-prolyl *cis*–*trans* isomerization (PPIase) activity and substrate protein binding. Chloroplast trigger-factor sequences have diversified from those of their bacterial orthologs and their molecular mechanism in plant organelles has been little investigated to date. Here, the crystal structure of the plastidic trigger factor from the green alga *Chlamydomonas reinhardtii* is presented at 2.6 Å resolution. Due to the high intramolecular flexibility of the protein, diffraction to this resolution was only achieved using a protein that lacked the N-terminal ribosome-binding domain. The eukaryotic trigger factor from *C. reinhardtii* exhibits a comparable dragon-shaped conformation to its bacterial counterpart. However, the C-terminal chaperone domain displays distinct charge distributions, with altered positioning of the helical arms and a specifically altered charge distribution along the surface responsible for substrate binding. While the PPIase domain shows a highly conserved structure compared with other PPIases, its rather weak activity and an unusual orientation towards the C-terminal domain points to specific adaptations of eukaryotic trigger factor for function in chloroplasts.

## Abstract 2

This study describes the structure of the chloroplast ribosome-associated molecular chaperone trigger factor at 2.6 Å resolution. It is shown that this eukaryotic trigger factor has evolved specific structural features in plants that are distinct from those of the bacterial homolog, contributing to a better understanding of co-translational protein folding in plastids.

## 1. Introduction

Molecular chaperones belong to a structurally diverse protein family which is dedicated to maintaining protein homeostasis in cells. Molecular chaperones assist their substrate or client proteins in *de novo* folding as well as in preventing substrates from misfolding or aggregation during stressful conditions (Balchin *et al.*, 2016 ▸). Chaperone-assisted protein folding starts co-translationally when newly synthesized polypeptides emerge from the ribosomal exit tunnel (Frydman, 2001 ▸; Koubek *et al.*, 2021 ▸; Pechmann *et al.*, 2013 ▸). In bacteria, the highly abundant ATP-independent molecular chaperone that associates with nascent polypeptides is termed trigger factor and is a well studied protein (Hoffmann *et al.*, 2010 ▸; Koubek *et al.*, 2021 ▸). Bacterial trigger factor (hereafter referred to as TF) has been shown to assist a broad spectrum of cytosolic, periplasmic and outer membrane substrates and associates with nascent polypeptides once the first 60 to 70 amino acids are accessible (Oh *et al.*, 2011 ▸). TF promotes the *de novo* folding of nascent polypeptides by shielding local partially folded conformations from premature distal interactions with other sections of the polypeptide (Mashaghi *et al.*, 2013 ▸). Besides the *bona fide* chaperone function on nascent polypeptides, TF has also been postulated to bind full-length proteins, thereby contributing to complex assembly and ribosome biogenesis (Hoffmann *et al.*, 2010 ▸; Liu *et al.*, 2005 ▸; Martinez-Hackert & Hendrickson, 2009 ▸; Saio *et al.*, 2014 ▸).

**Figure 1:**
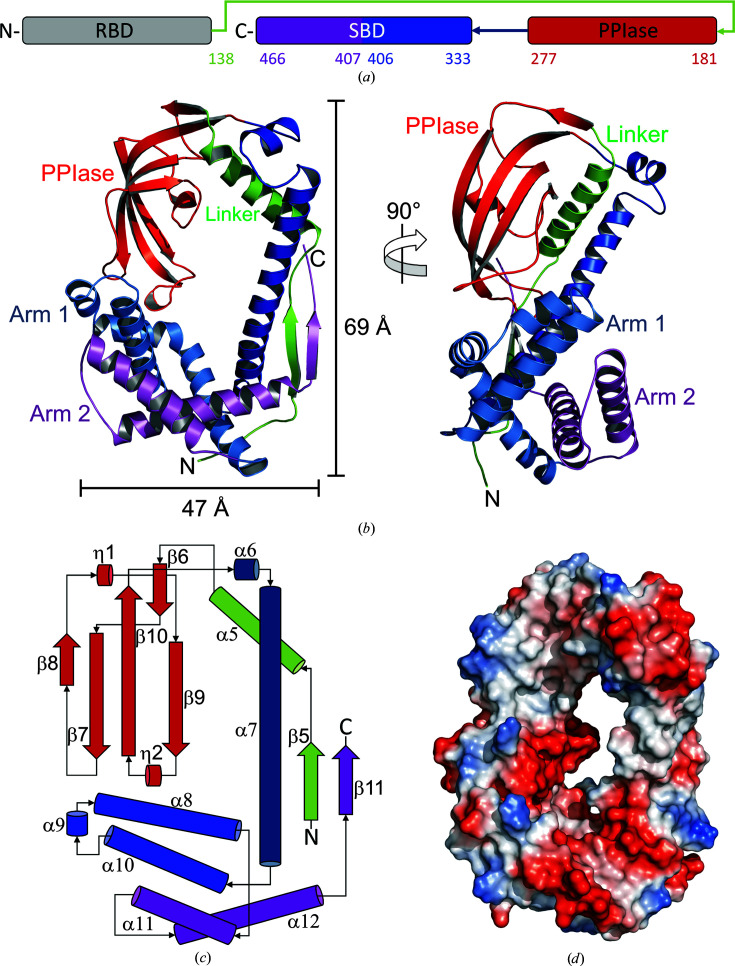
Crystal structure of *Cr*TIG1ΔRBD. (*a*) Domain assignment of full-length *Cr*TIG1. The missing N-terminal ribosome-binding domain (RBD) is colored gray, the long linker spanning the backbone is colored green, the PPIase domain is colored red and the long linker helix spanning from the PPIase domain to the C-terminal arms is colored blue. Arm 1 of the C-terminal chaperone activity domain (SBD) is colored light blue and arm 2 is colored magenta. (*b*) Ribbon presentation of the secondary-structure elements presented from the front view and from a side view rotated 90° counterclockwise [coloring is according to (*a*)]. (*c*) Scheme of the domain arrangement [coloring is according to (*a*)]. (*d*) Electrostatic surface potential from the front view (red indicates negative charge and blue indicates positive charge). The electrostatic surface potential was calculated with the *APBS* plugin from *PyMOL* (Baker *et al.*, 2001 ▸).

The *Escherichia coli* TF protein has a molecular weight of 48 kDa and has an elongated dragon-shaped conformation, bending over the ribosomal exit tunnel, which places the chaperone in an ideal position for embracing the emerging polypeptide (Deeng *et al.*, 2016 ▸; Ferbitz *et al.*, 2004 ▸). Ribosome interaction is modulated mainly via the N-terminal ribosome-binding domain (RBD; see Fig. 1 ▸ *a*, bottom), which forms the so-called tail of the dragon molecule. Ribosome binding is mainly mediated via the conserved GFR*x*G*xx*P signature motif and two surrounding α-helices of TF contacting ribosomal proteins Rpl23 and Rpl29 as well as domain III of the 23S ribosomal RNA (Ferbitz *et al.*, 2004 ▸; Kramer *et al.*, 2002 ▸). The middle domain in the linear sequence forms the active center of a dome-shaped peptidyl-prolyl *cis*–*trans* isomerase (PPIase) with sequence homology to FK506-binding proteins (FKBPs). The PPIase constitutes the so-called head, opposite the RBD, in the three-dimensional structure of TF. The PPIase allows TF to actively catalyze the *cis*–*trans* conversion of peptidyl-prolyl bonds *in vitro* (Hesterkamp *et al.*, 1996 ▸; Scholz *et al.*, 1997 ▸; Stoller *et al.*, 1995 ▸). However, the contribution of the PPIase to the chaperone function of TF is still under debate (Hoffmann *et al.*, 2010 ▸; Kramer *et al.*, 2004 ▸). PPIases can be classified into three subgroups: the FK506-binding proteins (FKBPs), cyclophilins (CYPs) and parvulins (He *et al.*, 2004 ▸; Vallone, 2005 ▸). FKBP proteins are often found in tandem with other modules in multidomain proteins. Their functions are described as scaffolders, holdases and foldases, with regulatory roles in protein folding, cell signaling, transcription and apoptosis (Dunyak & Gestwicki, 2016 ▸; Tong & Jiang, 2015 ▸). The main chaperone module of TF, here termed the substrate-binding domain (SBD), is formed by the C-terminal sequence of TF. This domain is mainly α-helical and shapes the back and two arms of the dragon-shaped molecule (Ferbitz *et al.*, 2004 ▸). The central cavity between the RBD and the two arms exhibits the peptide-binding capacity of the chaperone and is even capable of accommodating larger structural features of substrate proteins up to the size of the 14 kDa lysozyme (Ferbitz *et al.*, 2004 ▸). In recent years, structural information on several different prokaryotic TFs has been published for isolated TF (Ludlam *et al.*, 2004 ▸; Morgado *et al.*, 2017 ▸; Saio *et al.*, 2018 ▸; Ferbitz *et al.*, 2004 ▸; Martinez-Hackert & Hendrickson, 2009 ▸), TF in association with ribosomal proteins (Deeng *et al.*, 2016 ▸; Ferbitz *et al.*, 2004 ▸; Merz *et al.*, 2008 ▸; Baram *et al.*, 2005 ▸; Schlünzen *et al.*, 2005 ▸; Martinez-Hackert & Hendrickson, 2009 ▸) and with bound substrates (Saio *et al.*, 2014 ▸; Kawagoe *et al.*, 2018 ▸; Bhakta *et al.*, 2019 ▸) or single domains (Vogtherr *et al.*, 2002 ▸; Kawagoe *et al.*, 2018 ▸; Martinez-Hackert & Hendrickson, 2007 ▸; Kristensen & Gajhede, 2003 ▸), demonstrating that the overall domain architecture remains relatively conserved among the bacterial kingdom. However, the relative orientations of the individual TF domains display a remarkable variance between the different functional states and between organisms.

In eukaryotic cells, trigger factors are only found in the chloroplasts of plants (Ries *et al.*, 2017 ▸). Chloroplast trigger factors (hereafter referred to as TIG1s) display a remarkable sequence variance compared with prokaryotic TFs, while the typical organization of the three domains remains conserved. Recent low-resolution small-angle X-ray scattering (SAXS) analysis and *ab initio* modeling of TIG1 from *Chlamydomonas reinhardtii* (*Chlamydomonas*) and from *Arabidopsis thaliana* (*Arabidopsis)* demonstrated that the trigger-factor proteins have maintained the dragon-shaped conformation; however, their domain orientation seems to vary when compared with the conformation of TF from *E. coli* (*Ec*TF; Ries *et al.*, 2017 ▸). In addition, chloroplast TIG1 is not able to functionally replace the bacterial TF in *E. coli*, suggesting that the chaperone has evolved for its specific task in chloroplasts. However, ribosome association and aggregation preventing chaperone function is also preserved in the chloroplast ortholog (Rohr *et al.*, 2019 ▸).

The ribosome-binding signature motif of plastidic TIG1 is highly conserved within the land plants lineage (GFRP­G*xxx*P), whereas algal TIG1s show greater sequence variance in this region, with only the first glycine and the last proline being conserved in the motif. Interestingly, the algal trigger-factor binding site on the ribosomal Rpl23 protein shows equal sequence variance at this site, which points to co-evolution of the trigger-factor ribosome-binding interface in these phyla (Ries *et al.*, 2020 ▸; Rohr *et al.*, 2019 ▸). Disruption of bacterial and chloroplast trigger-factor genes results in no obvious growth defects under ambient conditions, but *tig1* mutants display retarded growth under heterotrophic conditions (Rohr *et al.*, 2019 ▸). However, the molecular mechanism of action of eukaryotic trigger factor and its substrate proteins during the biogenesis of chloroplast protein maturation are not known to date. A better understanding of this ortholog requires a deeper knowledge of the structural conformation of this protein, especially of the chaperone domain. We thus set out to solve the structure of *Chlamydomonas* TIG1 (*Cr*TIG1) at the atomic level. We resolved the X-ray structure of truncated *Cr*TIG1 with the PPIase domain and the C-terminal SBD. The structure shows an uncommon tilted PPIase domain which interacts intermolecularly with the SBD over a coiled-coil motif. The SBD does not contain the common ‘open arms’ features of the bacterial TF structure, but rather a conformation with aligned arms. The inner part of the cave built by the arms together with the PPIase domain shows a very negatively charged surface. The PPIase domain exhibits a low but measurable PPIase activity.

## 2. Methods

### 2.1. Protein purification

*Cr*TIG1 and *Ec*TF were expressed and purified as published previously (Ries *et al.*, 2017 ▸). Selenomethionine-substituted *Cr*TIG1 (hereafter referred to as *Cr*TIG1-SeMet) was produced in *E. coli* using a modified two-step protocol as described by Guerrero *et al.* (2001 ▸). A preculture was diluted in LB medium supplemented with ampicillin (100 µg ml^−1^), grown for 16 h, harvested and washed with sterile deionized water. The cells were then diluted in 2 l SelenoMethionine medium (Molecular Dimensions) supplemented with the same antibiotic and 40 µg ml^−1^ l-selenomethionine (Molecular Dimensions) to an OD_600_ of 1.0. After incubation for 30 min at 303 K, *Cr*TIG1-SeMet was expressed by the addition of 0.4 m*M* isopropyl β-d-1-thiogalactopyranoside for 17 h at 290 K. *Cr*TIG1-SeMet was purified as described for the native construct, except that 1 m*M* DTT was maintained in all buffers subsequent to elution from the chitin matrix.

### 2.2. Crystallization and crystal harvesting

For crystallization, native and selenomethionine-labeled *Cr*TIG1 were concentrated to 23 mg ml^−1^. Initial crystals were obtained in 96-well plates by the sitting-drop vapor-diffusion method at 291 K using the automated crystallization facility at the Department of Structural Biology, Saarland University (Müller & Lancaster, 2013 ▸). Equal amounts of protein solution (at protein concentrations of 23 and 11.5 mg ml^−1^, respectively) and reservoir solution were mixed and equilibrated against the reservoir solution. Crystals appeared between 10 and 90 days and were further optimized in 24-well plates using the hanging-drop vapor-diffusion method. Crystals were obtained at 277, 283 and 291 K in various crystallization conditions. Only crystals that grew at 283 and 291 K could be used for diffraction experiments; they diffracted to between 2.6 and 6 Å resolution. The crystals obtained at 277 K were very fragile and did not tolerate any handling or cryoprotectant. Crystals growing at 283 or 291 K tolerated glycerol over PEG 3350 as the best cryoprotectant. High-resolution structural information was obtained for native and selenomethionine-labeled proteins, which were crystallized under conditions containing magnesium sulfate (0.1–0.3 *M*) or ammonium sulfate (0.76–1.76 *M*) and MES buffer (pH 6.0–6.5), both with PEG 3350 as a precipitant.

### 2.3. Data collection, structure determination and refinement

The protein crystals were transferred to buffers consisting of the reservoir solution supplemented with 30% PEG 3350 or 30% glycerol for cryoprotection and were flash-cooled in liquid nitrogen. After initial diffraction experiments at the home source at the Department of Structural Biology (an Oxford Diffraction Nova system), X-ray diffraction data were collected at 100 K on beamline ID23-1 (Nurizzo *et al.*, 2006 ▸) at the European Synchrotron Radiation Facility (ESRF), Grénoble, France. Data were processed with either *XDS* (Kabsch, 2010 ▸) or *iMosflm* (Battye *et al.*, 2011 ▸) and were scaled with *XSCALE*, *SCALA* or *AIMLESS* (Evans, 2006 ▸) from the *CCP*4 software package (Potterton *et al.*, 2003 ▸) or processed with the automatic pipelines at the ESRF (Monaco *et al.*, 2013 ▸). The structure of *Cr*TIG1 was solved by phasing with selenomethionine using *SHELX* (Sheldrick, 2015 ▸). *Coot* (Emsley *et al.*, 2010 ▸) was used for manual rebuilding and completion of the model, and refinement was performed using *REFMAC*5 (Murshudov *et al.*, 2011 ▸). All data-collection and refinement statistics are summarized in Table 1 ▸. The coordinates and associated structure factors have been deposited at the Protein Data Bank as PDB entry 7zgi. Graphical representations of the structural model were created using *PyMOL* (DeLano, 2006 ▸). Further screening for crystals containing the full-length protein was performed on various beamlines at the ESRF and the Swiss Light Source (SLS).

### 2.4. Chymotrypsin-coupled PPIase activity assay

Peptidyl-prolyl *cis*–*trans* isomerization (PPIase) activity was measured with a protease-coupled assay (Fischer *et al.*, 1992 ▸) with modifications at 273 K. 50 µ*M* succinyl-Ala-Phe-Pro-Phe-*para*-nitro­anilide as the substrate peptide (Bachem Biochemica GmbH) was diluted in 35 m*M* HEPES buffer pH 7.6, 150 m*M* KCl and incubated with 0.1 mg ml^−1^ bovine α-chymotrypsin for 30 s, in which the pre-existing *trans* isoform of the substrate peptide was cleaved by chymotrypsin. To start the reaction, *Cr*TIG1 was added in the concentration range 1–25 µ*M*. Absorption at 395 nm was followed in a UV–Vis photometer (Ultraspec 2100 pro). *Ec*TF was used as a positive control and the spontaneous isomerization reaction without trigger factors was used as a negative control. Reaction rates were derived by nonlinear curve fitting to a first-order rate equation using *OriginPro* and were corrected for spontaneous isomerization.

### 2.5. Small-angle X-ray scattering experiments and data analysis

Small-angle X-ray scattering (SAX) data were collected on the BM29 beamline (Pernot *et al.*, 2013 ▸) at the ESRF with a PILATUS 1M detector (16.9 × 17.9 cm) at a wavelength of 0.9919 Å (12.5 keV) and a sample-to-detector distance of 2.867 m, corresponding to a *q*-range of 0.025–5 nm^−1^. Static measurements with three different protein concentrations of *Cr*TIG1 supplemented with 20-meric peptides from *Chlamydo­monas* chloroplast-encoded AtpB (the β subunit of the plastidic ATP synthase) and RbcL (the large subunit of Rubisco) were measured with 1 s exposure times per frame and ten frames per concentration at 293 K. The peptides were first dissolved in DMSO and added to the protein in a tenfold molar excess. Scattering by the corresponding buffer (20 m*M* Tris pH 7.5, 150 m*M* KCl, 1 m*M* DTT, 2% DMSO) was measured before and after one run, averaged and subtracted from the protein scattering. As a control, *Cr*TIG1 alone in buffer with the corresponding DMSO concentration was also measured. BSA standards were used to calibrate the *I*(0) values and the scattering of pure water was used to calibrate the intensity to absolute units (Orthaber *et al.*, 2000 ▸).

The data processing was performed with *ATSAS* 3.0.3 (Manalastas-Cantos *et al.*, 2021 ▸). The forward scattering *I*(0) and the radius of gyration *R*_g_ were evaluated with *PRIMUS* (Konarev *et al.*, 2003 ▸) using the Guinier approximation, assuming that for spherical particles at very small angles (*s* < 1.3/*R*_g_) the intensity is represented by *I*(*s*) = *I*(0)exp[−(*sR*_g_)^2/3^]. The distance distribution function *p*(*r*) and the maximum particle dimension (*D*_max_) were obtained using *GNOM* (Svergun, 1992 ▸). The frames collected from the static measurements were checked for radiation damage before averaging and buffer subtraction and those derived from different concentrations were merged.

### 2.6. Modeling of the full-length structure of *Cr*TIG1 into the SAXS shape

The protein structure of *Cr*TIG1ΔRBD and an *ab initio* model of the RBD were fitted manually into the SAXS shape derived from previous experiments (Ries *et al.*, 2017 ▸) using *UCSF Chimera* (Yang *et al.*, 2012 ▸). The *ab initio* model of the RBD was calculated with *I-TASSER* (Zhang, 2008 ▸) based on the *Ec*TF structure in PDB entry 2mlz (Saio *et al.*, 2014 ▸). The fit between the SAXS data and the structure was evaluated with *CRYSOL* (Svergun *et al.*, 1995 ▸).

## 3. Results

### 3.1. Crystallization trials of *Cr*TIG1

For crystallization of the chloroplast trigger-factor ortholog from *C. reinhardtii* (*Cr*TIG1), the mature protein, lacking the N-terminal 67 amino acids of the chloroplast transit peptide, was heterologously expressed in *E. coli* and purified, yielding protein without remaining affinity tags (Ries *et al.*, 2017 ▸). Data sets were collected from selenomethionine-labeled *Cr*TIG1 to 2.72 Å resolution with one molecule in the asymmetric unit and to 2.6 Å resolution with two molecules in the asymmetric unit in space group *C*121. The statistics for the data set at 2.6 Å resolution are summarized in Table 1 ▸. Although the structural domain arrangement was expected to be similar to that of the bacterial counterparts, molecular replacement with bacterial TF structures failed. Phasing with the anomalous scatterer selenium was successful and 11 of the 17 available atom positions were found and could be assigned to the sequence of the PPIase domain and the C-terminal substrate-binding domain (SBD). No suitable electron density was observed for the N-terminal ribosome-binding domain (RBD), which suggests that the RBD was absent in these high-resolution protein crystals. The model was further built by density modification and manual refinement. This initial structure was then used as a model to solve structures of *Cr*TIG1 from further data sets which were derived from several different native and selenomethionine-labeled *Cr*TIG1 protein preparations and diverse crystallization conditions. However, crystals containing the full-length protein exclusively diffracted anisotropically to diffraction limits lower than 6 Å in space groups *P*1, *P*2, *P*2_1_2_1_2_1_, *I*422 and *H*32. The diffraction limits of the crystals of the full-length protein could not be increased through variation of the incubation temperature, the use of different crystallization methods or seeding experiments. Small-angle X-ray scattering experiments on full-length *Cr*TIG1 and *At*TIG1 proteins point to highly flexible conformations (Ries *et al.*, 2017 ▸). Thus, chemical cross-linking was examined, as well as cocrystallization experiments with 20-meric peptides from the putative substrate proteins AtpB and RbcL, which had previously been identified by *in vitro* chaperone assays and peptide-spot assays, respectively (Rohr *et al.*, 2019 ▸). Due to the very fragile crystals of the full-length protein, the crystal-harvesting system (Zander *et al.*, 2016 ▸) and *in situ* crystallography at the ID30-B beamline (McCarthy *et al.*, 2018 ▸) at the ESRF were also tested. Through a combination of all these methods, the diffraction limits of full-length *Cr*TIG1 could be improved to 4 Å, which was still too low to solve the structure. Similar problems with anisotropy and lower diffraction limits of crystals of the full-length protein has also been described for *Thermotoga maritima* TF and is mainly caused by the high flexibility of the three domains (Martinez-Hackert & Hendrickson, 2009 ▸).

Dissolved crystals obtained from different crystallization conditions were investigated via SDS–PAGE and ESI mass spectrometry, which confirmed that the well diffracting crystals lack the N-terminal domain, whereas the crystals with low diffraction limits comprise the full-length protein. Of note, SDS–PAGE analyses indicated no degradation of the *Cr*TIG1 protein preparations when samples were stored over three months at 291 K (data not shown) and thus did not confirm the truncation of the RBD during crystallization. No putative protease cleavage site by *E. coli* proteases was predicted between the RBD and the PPIase domain using the ExPASy *PeptideCutter* tool and MEROPS (Gasteiger *et al.*, 2005 ▸; Rawlings *et al.*, 2018 ▸).

### 3.2. Unique domain arrangement of chloroplast trigger factor

The resulting atomic structure of *Cr*TIG1 lacks the first 137 amino acids of the mature protein (without the chloroplast signal peptide) and will be referred to here as *Cr*TIG1ΔRBD. Numbering of the amino-acid sequence starts with Ala1 and the secondary-structure elements are numbered according to the full-length *Ec*TF structure (Ferbitz *et al.*, 2004 ▸). Visible electron density starts with amino acid Val138 at the beginning of the linker region between the N-terminal RBD and the middle PPIase domain, with β5 being the first secondary-structure element. The protein resembles the dragon-like structure described for bacterial trigger factors, but without the tail (the RBD; Figs. 1 ▸ *a*–1 ▸ *c*). The C-terminal SBD is completely visible except for 15 amino acids at the C-terminus. The middle PPIase domain extends from Phe181 to Leu277. The C-terminal domain contains seven helices and one β-strand and can be divided into a long linker helix (Pro278–Asp332), arm 1 built from three helices (Met333–Leu406) and a second arm (Val407 to the end) with two helices. A long linker (Val138–Gly180) containing a β-strand followed by an α-helix connects the missing N-terminal domain and the PPIase domain and spans the complete backbone of the protein. The linker is stabilized via a parallel β-sheet built between the β-strand in the linker (β5) and the only β-strand at the C-terminus (β11) (Figs. 1 ▸ *b* and 1 ▸ *c*).

Calculation of the surface potential reveals an overall mixed pattern of acidic and basic patches in *Cr*TIG1ΔRBD, while the cradle formed by the PPIase domain and the C-terminal arms is mainly negatively charged (Fig. 1 ▸ *d*). Prominent hydrophobic patches are only found in the cavity of the PPIase domain and in arm 2 of the SBD (Supplementary Fig. S1).

**Figure 2:**
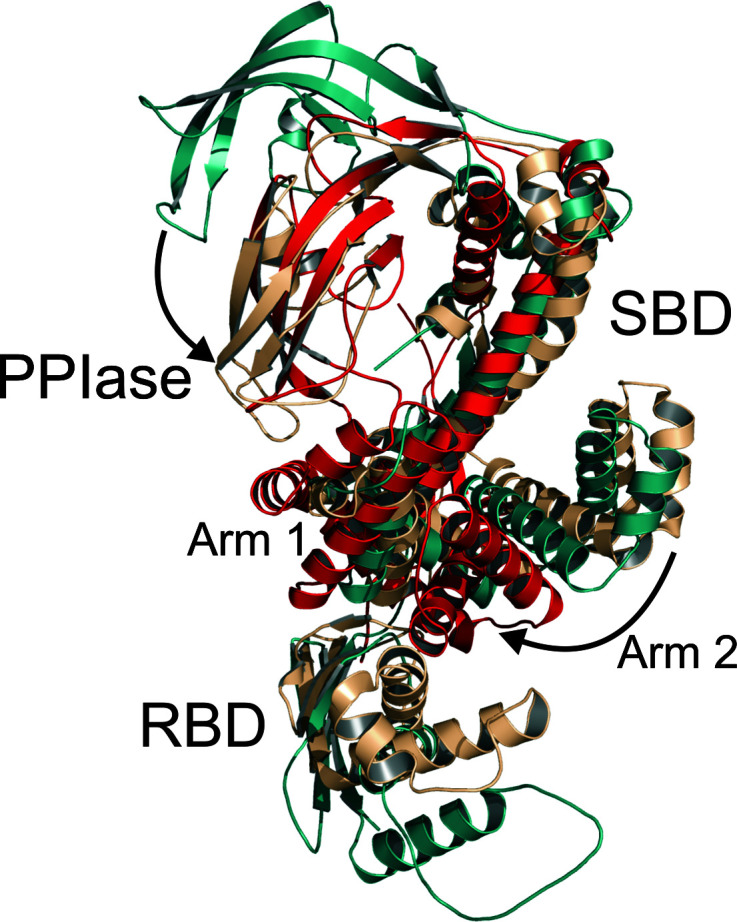
Structural alignment of *Cr*TIG1ΔRBD with *E. coli* trigger-factor structures in different conformations. *Cr*TIG1ΔRBD is colored red, monomeric *Ec*TF (PDB entry 1w26; Ferbitz *et al.*, 2004 ▸) is colored green and the solution structure of dimeric *Ec*TF (PDB entry 6d6s; Saio *et al.*, 2018 ▸) is colored beige. The most prominent movements, indicated by arrows, are observed for the PPIase domain and the relocation of arm 2 of the substrate-binding domain (SBD).

A global structural alignment of *Cr*TIG1ΔRBD with bacterial trigger factors was difficult due to the high flexibility of the domain arrangement, which explains the failure of our approach to solve the structure via molecular replacement based on all available prokaryotic TF structures. The closest structural homologue is the *E. coli* trigger factor, with a root-mean-square deviation (r.m.s.d.) of between 4.6 and 6.9 Å as determined with *DALI* (Holm, 2020 ▸; Fig. 2 ▸). Structural alignment of seven available full-length *Ec*TF structures shows that the PPIase domain can undergo major movement dependent on the binding or the interaction mode (substrate, ribosome or unbound; Supplementary Fig. S2). The most similar domain arrangement to that of the PPIase domain of *Cr*TIG1ΔRBD is found in the dimeric structures of *Ec*TF [PDB entries 5owi (Morgado *et al.*, 2017 ▸) and 6d6s (Saio *et al.*, 2018 ▸)], yet with clear differences. For example, the tips/loops of the *Cr*TIG1ΔRBD PPIase domain are tilted by 7.5 Å and an angle of 9° when compared with the dimeric *Ec*TF structure (PDB entry 6d6s; Saio *et al.*, 2018 ▸). In great contrast, the tilt is 35 Å and 48° between the positions of the PPIase domains in *Cr*TIG1ΔRBD and the structure of monomeric *Ec*TF (PDB entry 1w26; Ferbitz *et al.*, 2004 ▸; Fig. 2 ▸). The well characterized structures of TF from *Vibrio cholerae* (PDB entry 1t11; Ludlam *et al.*, 2004 ▸) and *T. maritima* (PDB entry 3gty; Martinez-Hackert & Hendrickson, 2009 ▸) are even less comparable, with the chloroplast ortholog showing an r.m.s.d. of 9.8 and 7.1 Å, respectively. Structural comparison of the different domains was also performed individually using the *DALI* server. Their characteristics and putative functions will be discussed separately.

### 3.3. The chloroplast trigger factor contains a conserved PPIase domain

**Figure 3:**
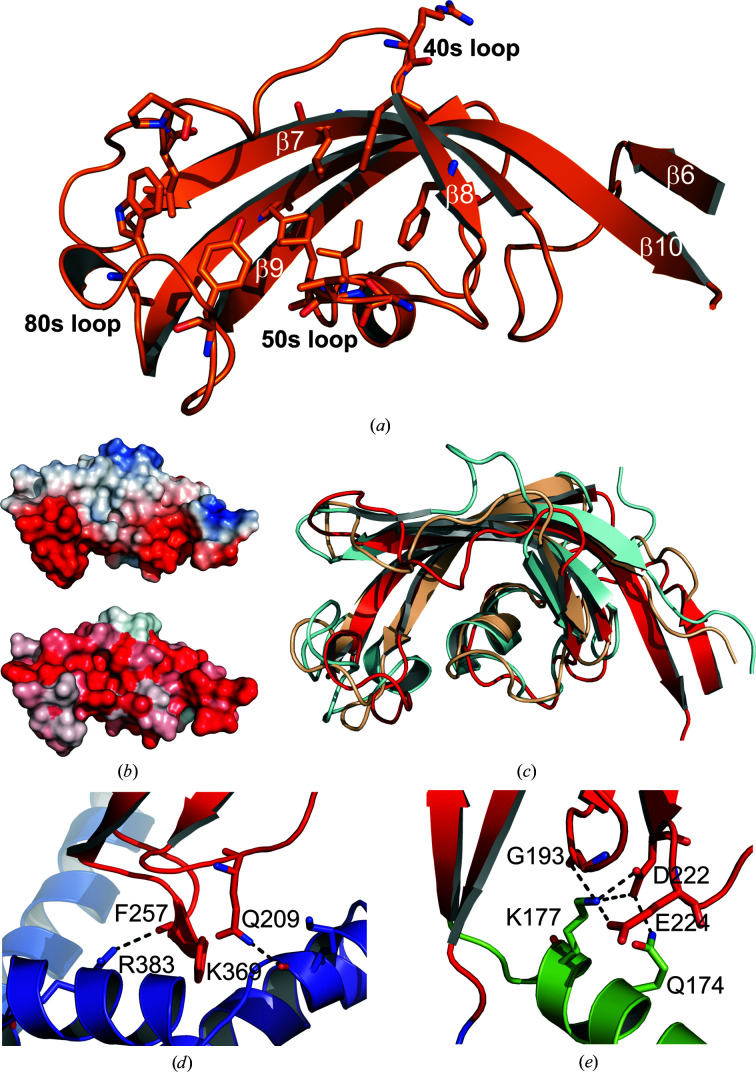
PPIase domain of *Cr*TIG1ΔRBD. (*a*) Secondary-structure elements are presented as ribbons. The loops are named according to the FKBP fold (Lücke & Weiwad, 2011 ▸). Conserved key residues involved in PPIase activity are shown as sticks. (*b*) Electrostatic surface potential (top) and hydrophobicity plot of the PPIase domain alone. The electrostatic surface potential was calculated with the *APBS* plugin from *PyMOL*. Red indicates negative charge and blue indicates positive charge. The hydrophobicity plot was generated with the *color_h* Python script in *PyMOL* (where red indicates the highest hydrophobicity; Eisenberg *et al.*, 1984 ▸). (*c*) Structural alignment of the PPIase domain of *Cr*TIG1ΔRBD (red) with those of *Ec*TF (beige; PDB entry 1w26; Ferbitz *et al.*, 2004 ▸) and the FKBP13–FK506 complex (light blue; PDB entry 4nnr; Schultz *et al.*, 1994 ▸). (*d*) Hydrogen bonds between the loops in the PPIase domain and arm 1 of the C-terminal domain (SBD). (*e*) Hydrogen bonds between the loops in the PPIase domain and the preceding linker helix.

The PPIase domain of *Cr*TIG1ΔRBD shows the typical conformation of FK506-binding proteins (FKBPs), consisting of a classical four-stranded antiparallel β-sheet forming a half β-barrel with two inserted 3_10_-helices. This sheet can be extended through a fifth β-strand (Van Duyne *et al.*, 1993 ▸). *Cr*TIG1ΔRBD also possesses a short fifth β-strand (β6 in our annotation) at the N-terminus. However, in contrast to the classical FKBP fold, this N-terminal β-strand does not extend the half β-barrel; instead, it builds a separate antiparallel β-sheet together with the C-terminal β-strand 10, which is very unusually elongated (Fig. 3 ▸ *a*). Prominent deviations from other FKBP proteins are further present in the 40s loop between β-strands 7 and 8 and in the 80s loop or so-called ‘flap’ (Fig. 3 ▸ *c*). The 40s loop often divides a β-strand into two parts, but in *Cr*TIG1ΔRBD only the second part of β-strand 8 is present. Instead, a long loop completely differently oriented to *Ec*TF is observed. In the 80s loop, an unusual 3_10_-helix is inserted at the tip of the loop. A second 3_10_-helix is inserted in the 50s loop. β-Strand 7 is extended and is surrounded by an extra-long loop between β-strands 6 and 7, causing a loop crossing. This motif is structurally rare since it is energetically unfavorable (Finkelstein *et al.*, 1993 ▸), and it needs a robust hydrogen-bond pattern in *Cr*TIG1 for stabilization. A comparable conformation is stabilized by disulfide bridges in human FKBP12 (Schultz *et al.*, 1994 ▸). In *Cr*TIG1ΔRBD, this huge loop is stabilized via hydrogen bonds to the 50s loop and the loop preceding β-strand 9. The half-barreled β-sheet builds a hydrophobic cavity lined with mostly aromatic amino acids. The surface of the PPIase represents an extreme bipolar charge distribution, with the back of the half-barrel being positively charged and the opposite site being extremely negatively charged, especially within the 80s and 50s loops (Fig. 3 ▸ *b*).

The PPIase domain, which forms the head of the dragon-like structure, is prominently tilted downwards in *Cr*TIG1ΔRBD towards the arms. This conformation is stabilized via several hydrogen bonds. Two hydrogen bonds are directed towards arm 1 of the C-terminal domain (Phe257 O to Arg383 N; Gln209 N to Lys369 O): one formed from the unusual 3_10_-helix in the flap of the PPIase domain and the second one from the neighboring loop after β-strand 7 (Fig. 3 ▸ *d*). Additional stabilizing hydrogen bonds are observed from Lys177 of the linker helix α5 to the prominent loop between β-strands 6 and 7 (to Glu224 and Asp222) and to Gly193 at the end of β-strand 8. Asp222 is also involved in a hydrogen bond to Gln174 in the linker helix (Fig. 3 ▸ *e*).

Within the PPIase domain of *Ec*TF, Glu178, Ile195, Phe198, Tyr221 and Phe233 are conserved and identified as key residues for PPIase activity (Liu *et al.*, 2010 ▸). The equivalent residues in the PPIase domain of *Cr*TIG1 are Pro212, Leu228, Ile230, Tyr253 and Val265 (Table 2 ▸, Supplementary Fig. S3). The best conserved region between the trigger factors is found in the region surrounding the position of Tyr253 in *Cr*TIG1.

The next structurally related neighbor of the *Cr*TIG1 PPIase domain is the respective domain in the conformation of monomeric *E. coli* TF (*Ec*TF; PDB entry 1w26; Ferbitz *et al.*, 2004 ▸), with an r.m.s.d. of 1.7 Å and a sequence identity of 22%, followed by other *Ec*TF structures. However, the PPIase domain of *Cr*TIG1 is also structurally related to peptidyl-prolyl *cis*–*trans* isomerases such as human FKBP13 (PDB entry 4nnr; Schultz *et al.*, 1994 ▸), FK506-binding protein 2 (PDB entry 2pbc; Structural Genomics Consortium, unpublished work) and SlyD (Quistgaard *et al.*, 2016 ▸), with r.m.s.d. values of between 2.0 and 2.4 Å and sequence identities of 12–17%. The next structural homologues in plants are *At*FKBP42 (PDB entry 2f4e; Weiergräber *et al.*, 2006 ▸) and the plastidic *At*FKBP13 (PDB entry 1u79; Gopalan *et al.*, 2004 ▸), with low sequence identities of 12% and 11%, respectively, and an r.m.s.d. of 2.4 Å (Supplementary Fig. S4). In contrast to *Cr*TIG1, these *Arabidopsis* PPIases differ in the number of β-strands and in the inlets in the 80s loop. Also, *At*FKBP13 and *At*FKBP42 contain disulfide bridges (Gopalan *et al.*, 2004 ▸). No such cysteines are present in the PPIase domain of *Cr*TIG1, again suggesting a different task of this domain in *Cr*TIG1 when compared with other plastidic PPIases.

### 3.4. *Cr*TIG1 exhibits a weak PPIase activity

**Figure 4:**
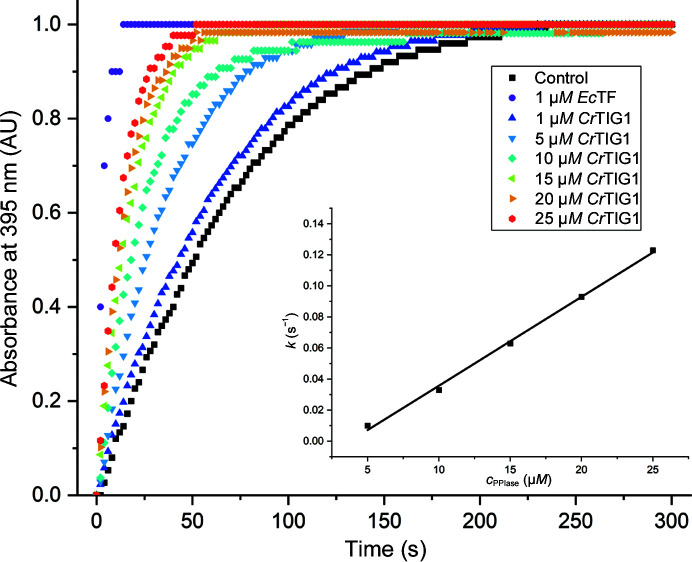
PPIase activity assay. The PPIase activities of *Cr*TIG1 and *Ec*TF were measured with a protease-coupled assay. The prolyl *cis*–*trans* isomerization of the tetrapeptide succinyl-Ala-Phe-Pro-Phe-*para*-nitroanilide as a substrate was monitored by measuring the absorbance changes at 395 nm at different *Cr*TIG1 concentrations. *Ec*TF was used as a positive control. The inset shows the change of the rate constants depending on the *Cr*TIG1 concentration.

The PPIase activity was measured with a protease-coupled enzyme assay in which *trans* isomer-specific proteolytic cleavage of the substrate succinyl-Ala-Phe-Pro-Phe-4-*para*-nitroanilide (suc-AFPF-pNA) was detected. The performance of the enzyme assay is based on the tetrapeptide assay described previously for *Ec*TF and is also used for determining the activities of chloroplast PPIases (Stoller *et al.*, 1995 ▸; Kramer *et al.*, 2004 ▸). *Ec*TF was used as a positive control and the spontaneous isomerization reaction without trigger factors was used as a negative control (Fig. 4 ▸). The catalytic activity *k*_cat_/*K*_m_ was determined to be 0.0057 µ*M* ^−1^ s^−1^ for *Cr*TIG1, which is 100-fold lower compared with the measured activity of *Ec*TF (0.64 µ*M* ^−1^ s^−1^). Previously determined *k*_cat_/*K*_m_ values for *Ec*TF with the suc-AFPF-pNA peptide as a substrate were 0.52 m*M* ^−1^ s^−1^ (Kramer *et al.*, 2004 ▸) and 0.74 µ*M* ^−1^ s^−1^ (Stoller *et al.*, 1995 ▸). Interestingly, *Cr*TIG1 only shows PPIase activity in an environment with a physiological salt concentration and therefore 150 m*M* KCl was used in the reaction buffer. This is consistent with a melting-point determination, in which *Cr*TIG1 and *At*TIG1 showed higher stability in buffers supplemented with increased salt conditions (Ries *et al.*, 2017 ▸).

### 3.5. Unique properties of the C-terminal domain of *Cr*TIG1

**Figure 5:**
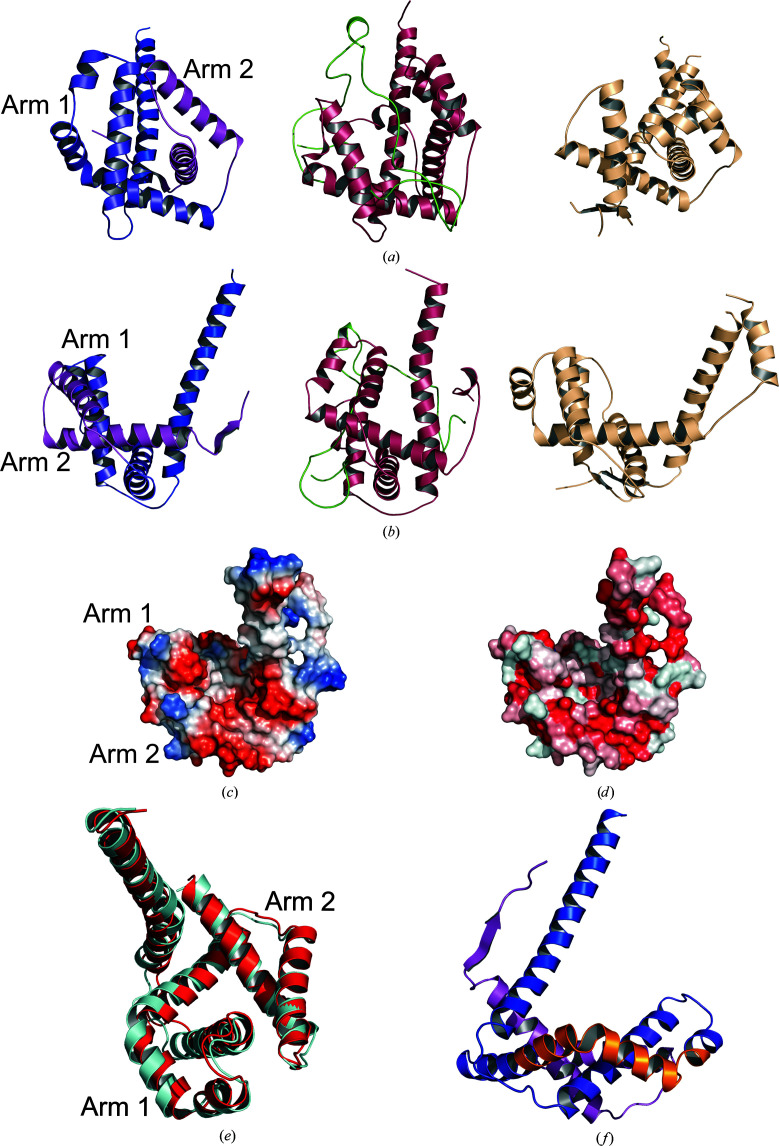
Structural features of the C-terminal substrate-binding domain (SBD). (*a*, *b*) Comparison of the C-terminal arms of *Cr*TIG1 with other chaperones in a front view (*a*) and a side view (*b*). Left, *Cr*TIG1; middle, *Ec*TF with bound substrate colored green (PDB entry 2mlz; Saio *et al.*, 2014 ▸); right, SurA complexed with peptide (PDB entry 2pv3; Xu *et al.*, 2007 ▸). (*c*) Electrostatic surface potential of the *Cr*TIG1 arms alone; view from the upper side. (*d*) Hydrophobicity plot of the *Cr*TIG1 arms alone; view from the upper side. (*e*) Alignment of chain *A* (red) and chain *B* (blue) found in the asymmetric unit of the *Cr*TIG1ΔRBD crystal. (*f*) Coiled-coil motif (colored in orange) in arm 1 of the C-terminal SBD.

The C-terminal domain of *Cr*TIG1 stands out by its unique features and differently arranged arms, which are not found in bacterial trigger factors. The arms are oriented towards each other and do not show the prominent ‘open arms’ conformation of the bacterial trigger factors, which build a cavity for substrate protein binding (Saio *et al.*, 2014 ▸; Figs. 5 ▸ *a* and 5 ▸ *b*). The thus built lap in *Cr*TIG1 mostly contains negative charged amino acids and is mainly surrounded by hydrophobic amino acids (Figs. 5 ▸ *c* and 5 ▸ *d*). The conformation of the SBD structure most closely resembles the structures of the *E. coli* trigger factor [PDB entries 1w26 (Ferbitz *et al.*, 2004 ▸) and 2mlz (Saio *et al.*, 2014 ▸)] and the periplasmic chaperone SurA (Xu *et al.*, 2007 ▸) (Figs. 5 ▸ *a* and 5 ▸ *b*). In *Cr*TIG1 the location of arm 1 is comparable to the respective arm of *Ec*TF, but arm 2 is arranged differently, with a distance of 24 Å and an angle of 27° with monomeric *Ec*TF (PDB entry 1w26; Ferbitz *et al.*, 2004 ▸) and a distance of 39 Å and an angle of 54° with the dimeric *Ec*TF NMR structure (PDB entry 6d6s; Saio *et al.*, 2018 ▸) (Fig. 2 ▸). Comparison of both chains in the asymmetric unit of the crystal structure shows differences in the position of arm 1 but smaller differences in arm 2. Also, the backbone helix has a kink in chain *B* compared with chain *A* (Fig. 5 ▸ *e*). Helix α10 in arm 1 is unusually kinked and contains a coiled-coil motif at amino acids 360–392 as predicted by the *SMART* server (Letunic *et al.*, 2021 ▸; Fig. 5 ▸ *f*).

### 3.6. *Cr*TIG1 shows a more compact form with a tilted PPIase domain and closed arms

**Figure 6:**
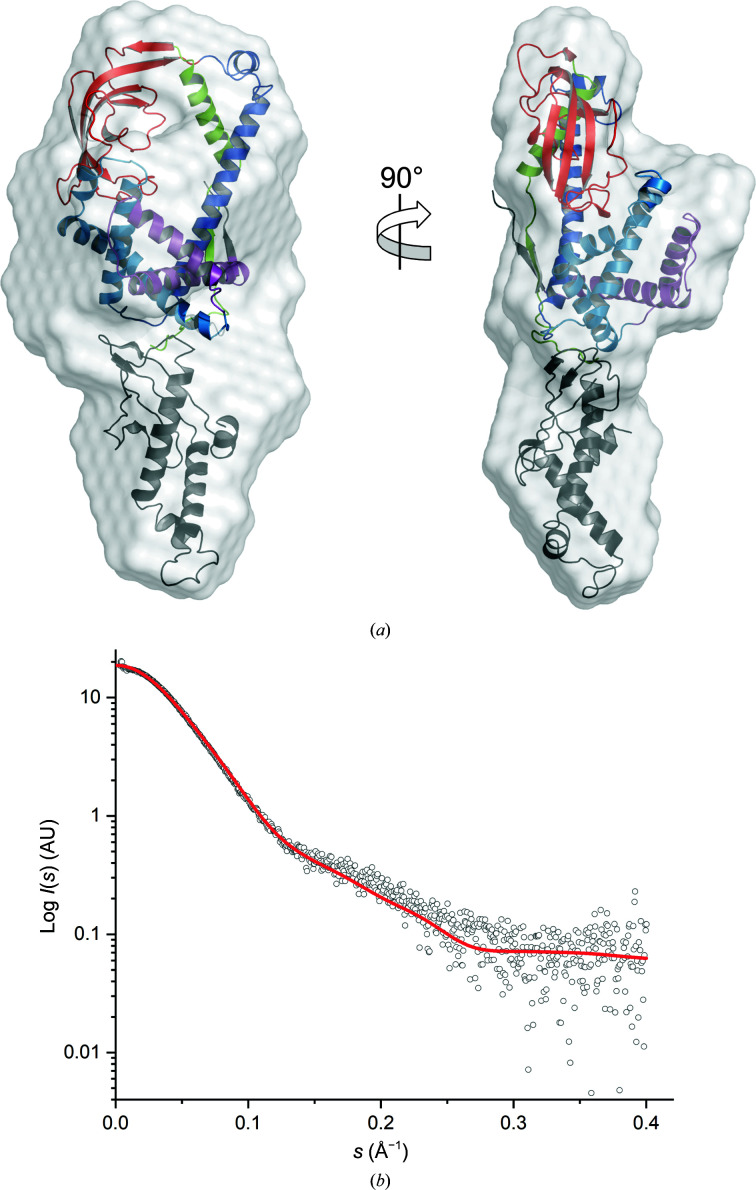
Fitting of the rigid-body model of the RBD (in gray) and the X-ray structure model of *Cr*TIG1ΔRBD into the SAXS shape first described by Ries *et al.* (2017 ▸). (*a*) The secondary-structure elements are drawn as ribbons; the SAXS bead model is shown in surface representation. The coloring of the domains is according to Fig. 1 ▸(*a*). (*b*) *CRYSOL* fit (χ^2^ = 1.88) of the assembled *Cr*TIG1 structure to the SAXS scattering data.

To determine whether *Cr*TIG1ΔRBD might have artificial arrangements caused by truncation of the N-terminal domain, we fitted the X-ray structure together with the rigid-body model of the N-terminal RBD into the SAXS bead model of full-length *Cr*TIG1, which we had published previously (Ries *et al.*, 2017 ▸; Fig. 6 ▸). The final model was evaluated with *CRYSOL* (χ^2^ = 1.88), which indicates a good alignment.

While the N-terminal domain might have more latitude to move in the SAXS envelope, the PPIase domain is likewise tilted down to the SBD and the arms are still in the closed conformation. There seems to be no free space for an open conformation of the PPIase domain and the arms of the SBD. This indicates that full-length *Cr*TIG1 in solution has a comparable closed conformation with interaction between the PPIase domain and the arms as in the crystal structure.

We also investigated the conformation of *Cr*TIG1 with peptides from the putative interaction partners AtpB (the β subunit of the plastidic ATP synthase) and RbcL (the large subunit of Rubisco) as extracted from peptide-spotting arrays (Rohr *et al.*, 2019 ▸). The structural parameters point to slight conformational changes but no relocation of domains, as shown for *Ec*TF structures (Supplementary Fig. S5). Taken together, the structure of *Cr*TIG1ΔRBD resembles the bacterial counterparts but displays unique features such as an unusual orientation of the head and arms and also a special surface-charge pattern.

## 4. Discussion

Over the past 15 years, numerous structures of bacterial trigger factors have been obtained by NMR, X-ray crystallography and cryo-EM, with *Ec*TF being by far the best investigated trigger factor and possibly one of the best-understood molecular chaperones in the literature. In contrast, no structural information on chloroplast trigger factors had been obtained until now despite their unique presence in eukary­otic cells and detectable functional deviations from their prokaryotic origin (Rohr *et al.*, 2019 ▸). Comparison of our chloroplast trigger-factor structure with all full-length structures of bacterial trigger factors from *E. coli*, *T. maritima* and *V. cholerae* revealed the very flexible arrangement of the three domains within the molecule. This agrees with a previous SAXS analysis of *Cr*TIG1 and *At*TIG1, showing that both of these proteins are likewise intrinsically flexible (Ries *et al.*, 2017 ▸).

### 4.1. The PPIase domain of chloroplast trigger factor

In the crystal structure of *Cr*TIG1ΔRBD and in the SAXS model of full-length *Cr*TIG1 in solution, the PPIase domain is very tilted down and connects to arm 1 of the SBD. Similar behavior is found in the structures of dimeric *Ec*TF in solution (Morgado *et al.*, 2017 ▸; Saio *et al.*, 2018 ▸) and in the X-ray structure of a trigger-factor dimer from *V. cholerae* (Ludlam *et al.*, 2004 ▸). Dimerization appears to be a molecular strategy to control the activity of the trigger factor at the ribosome and in its free state. The dimeric TF associates faster with proteins and exhibits stronger anti-aggregation and holdase activity than the monomeric TF (Patzelt *et al.*, 2002 ▸; Morgado *et al.*, 2017 ▸; Saio *et al.*, 2018 ▸). In the asymmetric unit of the crystal packing of *Cr*TIG1ΔRBD two molecules were found, but analysis with the *PISA* server (Krissinel & Henrick, 2007 ▸) indicated no formation of a dimeric complex. This goes in hand with previous small-angle X-ray scattering results and size-exclusion chromatography experiments, which showed that *Cr*TIG1 is mainly monomeric in solution (Ries *et al.*, 2017 ▸). However, it cannot be excluded that the interfaces might be crystal-packing contacts.

The conformation of the PPIase domain of chloroplast *Cr*TIG1 shows remarkable overall conservation compared with other PPIase domains across kingdoms. Also, the binding pocket in the inner cavity of the half barrel contains nonpolar and aromatic amino acids which are conserved among FKBPs. However, we observed several structural features that seem to be unique to the PPIase domain of *Cr*TIG1 when compared with the respective domains of other trigger factors or FKBP proteins. The similarities and differences of key residues in the active site of the *Cr*TIG1 PPIase domain, which are related to PPIase activity, are summarized in Table 2 ▸. The structural elements and sequences in the 40s and 80s loops are particularly important for substrate recognition by FKBPs and protein–protein interactions, as demonstrated for the interplay between hFKBP12 and FRAP (FKBP-rapamycin-associated protein; Choi *et al.*, 1996 ▸; Lücke & Weiwad, 2011 ▸). Interestingly, the 40s loop of *Cr*TIG1 does not shows clear homology to either human FKBP13 or to the *Ec*TF PPIase domain as the next structural homologues. An intramolecular hydrogen bond between Tyr27 and Asp38 inside the binding pocket is described in human FKBP12 and other active PPIases, but this bond is missing in *Ec*TF and *Cr*TIG1. In *Cr*TIG1 the corresponding part preceding the 40s loop is disordered and only contains nonpolar residues. It is stabilized through a hydrogen-bond network in the peptide backbone to the neighboring β-strand 7. Likewise, the 80s loop or ‘flap’ of *Cr*TIG1 shows structural particularities, being of equal length but differently oriented. The 50s loop seems to be responsible for substrate specificity (Weiergräber *et al.*, 2006 ▸). The backbone confirmation of this loop appears to play the most significant role for PPIase activity in substrate binding and is stabilized via a structural water molecule (Szep *et al.*, 2009 ▸). In *Cr*TIG1, Asp226 stabilizes this loop instead of a water molecule. Also, the amino-acid composition in this stretch is important for activity (Gollan *et al.*, 2011 ▸). In *Cr*TIG1, the sequence F^221^DTEAD^226^V^227^L^228^ indicates PPIase activity. Furthermore, the complete PPIase domain of *Cr*TIG1 shows an entirely reverted distribution of its surface charge. While hFKBP12 and *Ec*TF show a mixed pattern of negative and positive patches in this segment, the *Cr*TIG1 PPIase domain has a negative bottom and a positive backbone. In the altered charge distribution, the ‘flap’ stands out with its very negative polarity, a feature that is not observed for *Ec*TF or hFKBP12.

However, neither the substitution of the conserved key active-site residues within the hydrophobic pocket of the PPIase domain of *Cr*TIG1 nor the structural differences in the loops necessarily point to loss of its PPIase activity (Lücke & Weiwad, 2011 ▸). Also, comparisons of the active-site residues in *Cr*TIG1 with *Ec*TF and related PPIase domains as well as the amino-acid sequence in the 50s loop indicate an active domain. Indeed, we measured a PPIase activity of *Cr*TIG1 of 0.0057 µ*M* ^−1^ s^−1^, which is 100-fold lower than that of *Ec*TF with the substrate, but still measurable and within the range of other plant FKBP proteins (Singh *et al.*, 2020 ▸). An FK506-binding protein with comparable low-level PPIase activity (*k*_cat_/*K*_m_ = 0.021 µ*M* ^−1^ s^−1^) is *At*FKBP20-2, which is involved in the accumulation of the PSII supercomplex in *Arabidopsis* (Lima *et al.*, 2006 ▸). Thus, the chloroplast trigger factor possesses a conserved, albeit adapted, PPIase domain with measurable activity. However, the contribution of this domain to the function of the chaperone remains elusive, and it cannot be excluded at this point that the PPIase domain might have evolved towards rather atypical substrates.

### 4.2. The substrate-binding interfaces of chloroplast trigger factor

*Ec*TF exhibits a broad substrate spectrum and seems to bind several hundred nascent polypeptides during their synthesis (Oh *et al.*, 2011 ▸). In contrast, fewer than 100 different proteins are expressed from the chloroplast genome and *in vitro* studies indicate less general substrate specificity of *Cr*TIG1 (Rohr *et al.*, 2019 ▸). *Ec*TF preferably binds peptides with a positive net charge and recognizes aromatic and basic amino-acid residues in peptide substrates (Patzelt *et al.*, 2001 ▸). In *Cr*TIG1 the distribution of the electrostatic and hydrophobic potential shows a mixed pattern on the backbone of the protein, but the inner core between the PPIase domain, the arms and the linker is particularly negatively charged. The amino acids in the peptides identified to bind to both of the eukaryotic trigger factors *Cr*TIG1 and *At*TIG1 described in Rohr *et al.* (2019 ▸) have no enrichment in basic or acidic peptides (11%/12%), with an average content of 34% nonpolar residues. The content of aromatic residues in these peptides is 12%, while nonbinding peptides mostly lack aromatic amino acids. Regarding peptides exclusively bound by *Cr*TIG1, the content of non­polar residues increases to 40% and the ratio of basic/acidic peptides increases to 14%/9%.

The substrate-binding domain of *Cr*TIG1 shows structural homology to SurA. The chaperone activity of SurA in the periplasm of *E. coli* is essential for outer membrane protein biogenesis (Sklar *et al.*, 2007 ▸). Ribosome profiling for *Ec*TF showed that β-barrel outer-membrane proteins were the most prominent substrates (Oh *et al.*, 2011 ▸). *Cr*TIG1 is mainly localized in the stroma and it still remains to be shown whether it contributes to nascent polypeptide maturation of thylakoid membrane proteins, which would explain the similar structural features to SurA.

Arm 1 of the SBD forms an unusual coiled-coil motif and also appears to be more flexible than arm 2. Coiled-coil motifs in chaperones have been discussed to mediate the binding of unfolded proteins (Martin *et al.*, 2004 ▸). Archaeal prefoldin (PFD), for example, interacts with nonfolded proteins via a coiled-coil network (Siegert *et al.*, 2000 ▸). The role of the putative coiled-coil motif in *Cr*TIG1 remains unclear, but it might be the anchor point or interaction surface for unfolded peptide chains in the active state of the trigger factor. In the inactive state, the coiled-coil motif may stabilize the PPIase domain. It remains to be shown whether formation of the coiled coil may be absent in the ribosome-associated chloro­plast TIG1 and thus promote nascent polypeptide binding. Also, for *Ec*TF, MD simulation shows a more compact structure in solution and a framework of intermolecular inter­actions between the head and arm 1 (Singhal *et al.*, 2013 ▸).

## 5. Conclusion

The crystal structure of a eukaryotic trigger factor is presented. The structure of the trigger factor of the green alga *C. reinhardtii* resembles that of prokaryotic trigger factors, but with different domain orientations as well as an unusual conformation of the arms in the SBD and also uncommon intramolecular domain interactions. While bacterial trigger factors exhibit promiscuous substrate binding and high plasticity, the more distinct negative and hydrophobic patches of *Cr*TIG1ΔRBD in the cradle of the arms and the PPIase domain are compatible with a more specialized function in the chloroplast. Further studies specifically aimed at substrate identification of chloroplast TIG1, such as selective ribosome profiling, are required to precisely understand its role during protein biogenesis. However, the present structure already indicates specific structural adaptation for the binding of proteins in plant organelles. Further, structural availability of the RBD would allow the nature of the ribosome inter­action in chloroplasts to be modelled.

## 6. Related literature

The following references are cited in the supporting information for this article: Gouet *et al.* (1999 ▸) and Madeira *et al.* (2019 ▸).

## Supplementary Material

PDB reference: chloroplast trigger factor, 7zgi

Supplementary Figures. DOI: 10.1107/S2059798322009068/jv5013sup1.pdf

## Figures and Tables

**Table 1:** Data-collection and refinement statistics Values in parentheses are for the highest resolution shell.

PDB code	7zgi
Data collection	
Beamline	ID23-1, ESRF
Space group	*C*121
*a*, *b*, *c* (Å)	175.29, 69.88, 106.04
α, β, γ (°)	90, 110.99, 90
Wavelength (Å)	0.97779
Resolution (Å)	54.31–2.60 (2.70–2.60)
No. of observations	244831 (30623)
No. of unique reflections	37064 (4515)
Completeness (%)	99.8 (99.9)
Anomalous completeness (%)	99.4 (99.4)
Multiplicity	6.6 (6.8)
〈*I*/σ(*I*)〉	10.2 (3.0)
*R*_merge_ (%)	9.4 (54.8)
*R*_meas_ † (%)	11.2 (65.1)
*R*_p.i.m._ ‡ (%)	6.0 (34.8)
CC_1/2_	0.996 (0.941)
Wilson *B* factor (Å^2^)	49.1
Refinement	
*R*_cryst_ §/*R*_free_ ¶ (%)	23.5/28.3
No. of molecules in the asymmetric unit	2
Residues included in the model [chain]	Pro140–Ala466 [*A*]/Val138–Ala466 [*B*]
Total No. of protein atoms	5207
Water molecules	26
Ligands	Sulfate, polyethylene glycol
Overall *B* factor (Å^2^)	57.9
Average *B* factor, protein chain *A*/*B* (Å^2^)	59.2/56.2
Average *B* factor, waters (Å^2^)	39.4
Ramachandran outliers (%)	
Favored	96
Allowed	4
Outliers	0
R.m.s.d., bond lengths (Å)	0.011
R.m.s.d., bond angles (°)	1.59

† *R*_meas_ = 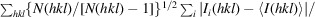 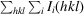 .

‡ *R*_p.i.m._ = 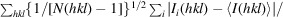 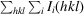 .

§ *R*_cryst_ = 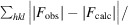 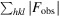 .

¶ Calculation of *R*_free_ was performed analogously to that of *R*_cryst_ but using 5% of randomly chosen reflections.

**Table 2:** Key residues in the active sites of the PPIase domains in the trigger factors *Ec*TF and *Cr*TIG1 based on the structure of human FKB12 (hFKBP12; Lücke & Weiwad, 2011 ▸) as a prototypical FK506-binding protein

hFKBP12	*Ec*TF	*Cr*TIG1	Possible effect on PPIase activity
Tyr27	Phe168	Leu200	Negative
Phe137	Phe177	Leu211	?
Asp38	Glu178	Pro212	?
Arg46	Asp184	Arg218	?
Phe147	Phe185	Phe219	Positive
Phe149	Leu187	Phe221	Positive
Glu55	Arg193	Asp226	?
Val56	Met194	Val227	Positive
Ile57	Ile195	Leu228	Positive
Trp60	Phe198	Ile230	Negative
Tyr83	Tyr221	Tyr253	Positive
His88	—	—	—
Ile92	Leu226	Trp258	?
Phe1100	Phe1233	Val265	?
