# Supplementary material for: Structural features of chloroplast trigger factor determined at 2.6 Å resolution
Source: Acta Crystallogr D Struct Biol. 2022 Sep 27;78(Pt 10):1259–72. doi: 10.1107/S2059798322009068 (PMC9527764; doi:10.1107/S2059798322009068)
Supplement: Supplementary file 1 [file d-78-01259-sup1.pdf]

STRUCTURAL BIOLOGY

Volume 78 (2022)

Supporting information for article:

Structural features of chloroplast trigger factor determined at 2.6 Å resolution

Yvonne Carius, Fabian Ries, Karin Gries, Oliver Trentmann, C. Roy D. Lancaster and Felix Willmund

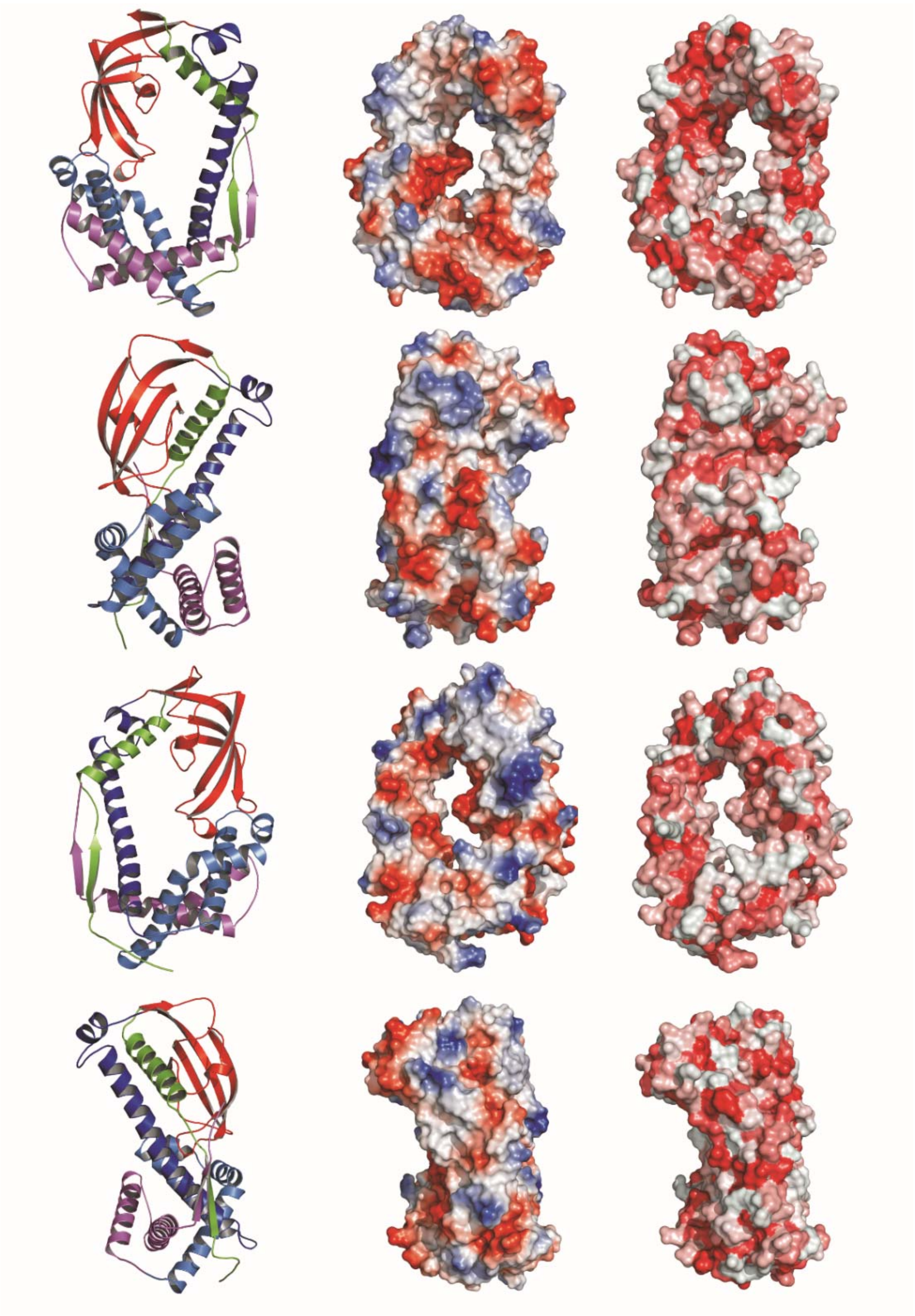

Figure S1 Overall structure of *Cr*TIG1ΔRBD shown from 4 view sides (first row), the corresponding electrostatic surface potential (middle row) and the distribution of hydrophobic residue pattern (right row). Every view is rotated of 90° counterclockwise. The secondary-structure elements are shown as ribbons. The PPIase domain is colored in red, the preceding linker in green, the long linker helix spanning from PPIase domain to the C-terminal arms in blue. The arm 1 of the C-terminal chaperone activity domain is painted in light blue, arm 2 in magenta. The electrostatic surface potential was calculated with the APBS plugin from PyMol (red indicates negative and blue positive charge, respectively). Hydrophobicity plot was done with the color_h python script in PyMol (red means highest hydrophobicity).

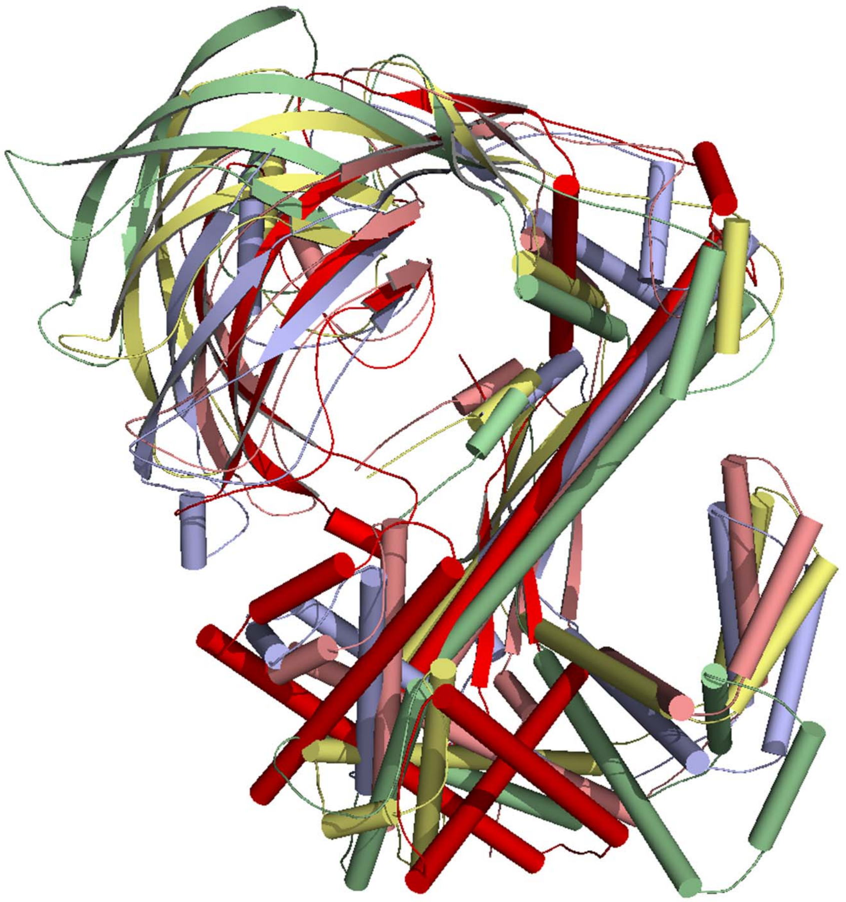

Figure S2 Structural alignment of *Cr*TIG1ΔRBD (red) with published *Ec*TF structures covering most possible conformations: monomeric *Ec*TF (green, 1W26 (Ferbitz *et al.*, 2004)), in complex with unfolded PhoA (yellow, 2MLX (Saio *et al.*, 2014)), dimeric structure in solution (pink, 5OWI (Morgado *et al.*, 2017)), dimeric solution structure (blue, 6D6S (Saio *et al.*, 2018)). For better clarity, the RBD is not shown.

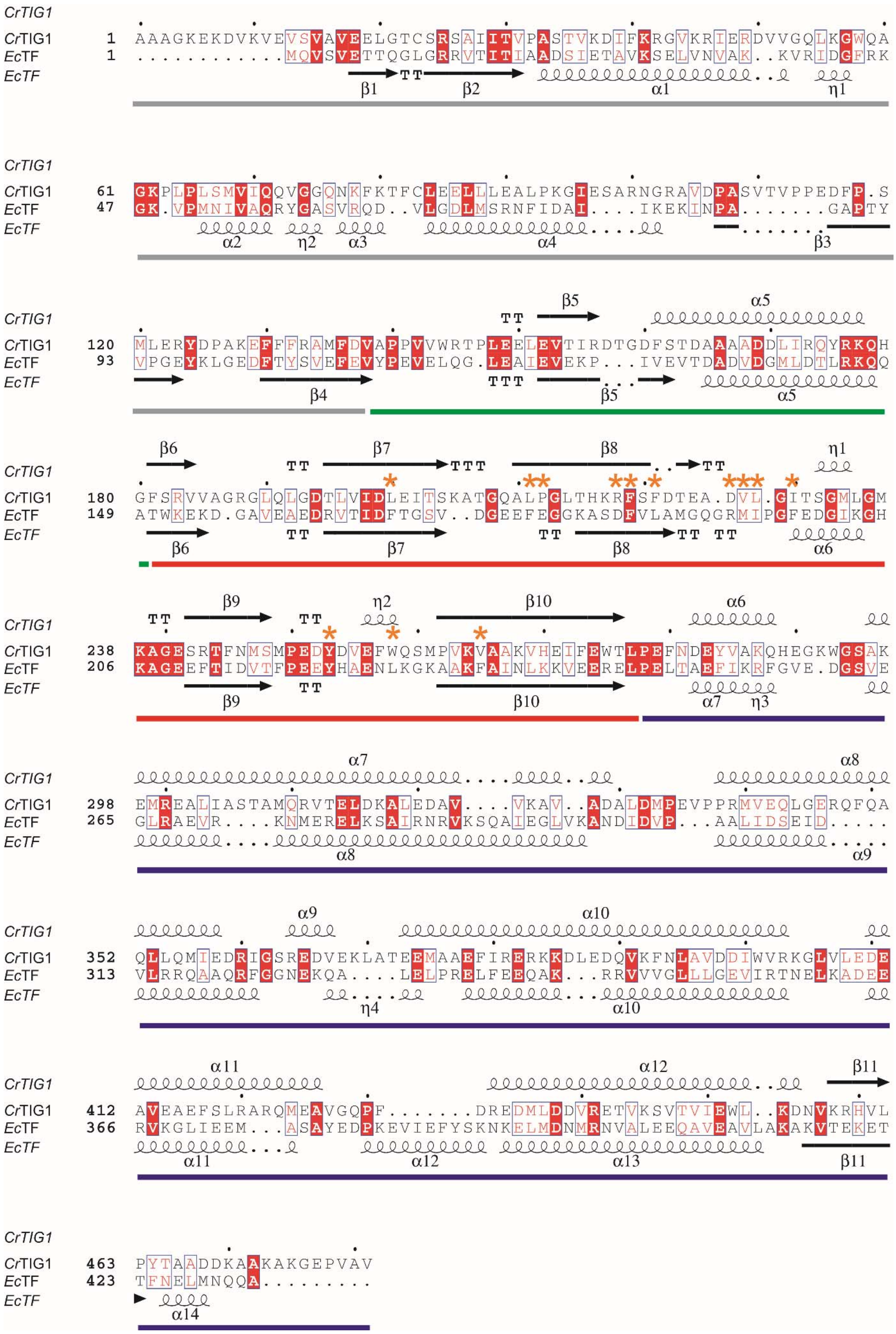

Figure S3 Structural-based sequence alignment between *Cr*TIG1 and *Ec*TF (PDB entry 1W26 (Ferbitz *et al.*, 2004)) performed with the program Clustal Omega (Madeira *et al.*, 2019). The final figure with the secondary-structure elements was prepared with the server ESPript 3.0 (Gouet *et al.*, 1999). The colored bars show the domain barrier (grey: RBD, green: linker, red: PPIase, blue SBD). The orange asterisks indicate conserved key residues involved in PPIase activity.

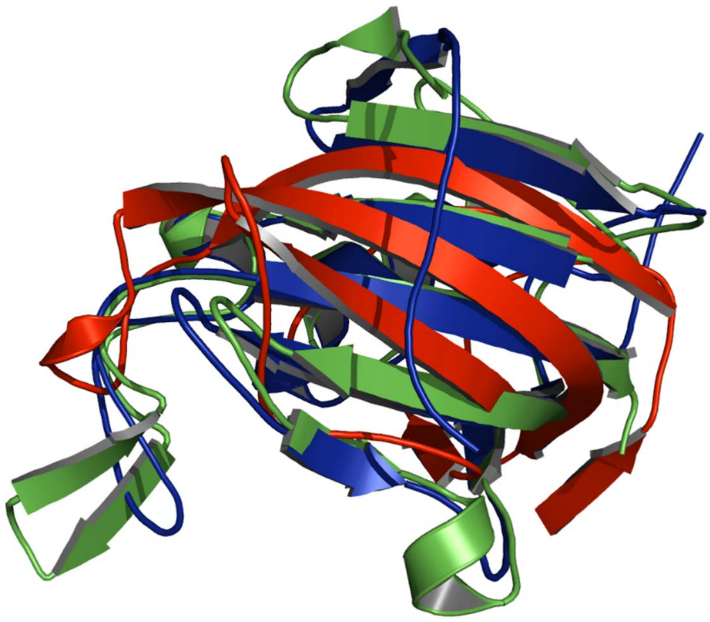

Figure S4 Alignment of *Cr*TIG1 PPIase domain (red) with *At*FKBP13 (green) and *At*FKBP42 (blue).

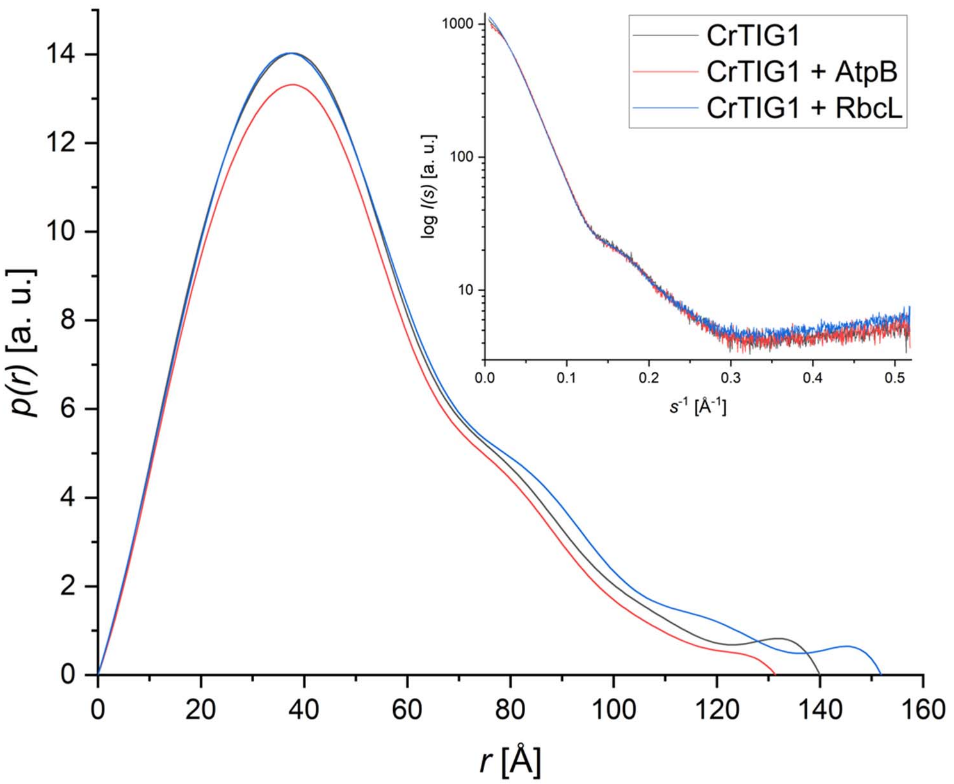

Figure S5 SAXS analysis of *Cr*TIG1 bound to peptides of AtpB and RbcL. The applied peptides were identified in a previous peptide spot-assay (Rohr *et al.*, 2019). The inlet shows the 1D scattering curves corresponding to the Pair-distribution functions in the main picture.
